# Use of the *FMR1* Gene Methylation Status to Assess the X-Chromosome Inactivation Pattern: A Stepwise Analysis

**DOI:** 10.3390/genes13030419

**Published:** 2022-02-25

**Authors:** Bárbara Rodrigues, Ana Gonçalves, Vanessa Sousa, Nuno Maia, Isabel Marques, Emídio Vale-Fernandes, Rosário Santos, António J. A. Nogueira, Paula Jorge

**Affiliations:** 1 Molecular Genetics Unit, Centro de Genética Médica Dr. Jacinto Magalhães (CGM), Centro Hospitalar Universitário do Porto (CHUPorto), 4099-028 Porto, Portugal; be_rodrigues_@hotmail.com (B.R.); ana.goncalves@chporto.min-saude.pt (A.G.); vanessa_sousa4d@hotmail.com (V.S.); nuno.maia@chporto.min-saude.pt (N.M.); isabel.marques@chporto.min-saude.pt (I.M.); rosario.santos.cgm@chporto.min-saude.pt (R.S.); 2 UMIB—Unit for Multidisciplinary Research in Biomedicine, ICBAS—School of Medicine and Biomedical Sciences, University of Porto, 4050-313 Porto, Portugal; emidiovalefernandes.pma@chporto.min-saude.pt; 3 ITR—Laboratory for Integrative and Translational Research in Population Health, 4050-313 Porto, Portugal; 4 Centre for Medically Assisted Procreation/Public Gamete Bank, Centro Materno-Infantil do Norte Dr. Albino Aroso (CMIN), Centro Hospitalar Universitário do Porto (CHUPorto), 4050-651 Porto, Portugal; 5 CESAM—Center for Environmental and Marine Studies, Department of Biology, University of Aveiro, 3810-193 Aveiro, Portugal; antonio.nogueira@ua.pt

**Keywords:** X-chromosome inactivation pattern, HUMARA, *FMR1* methylation status, methylation PCR

## Abstract

X-chromosome inactivation (XCI) is a developmental process to compensate the imbalance in the dosage of X-chromosomal genes in females. A skewing of the XCI pattern may suggest a carrier status for an X-linked disease or explain the presence of a severe phenotype. In these cases, it is important to determine the XCI pattern, conventionally using the gold standard Human Androgen-Receptor Assay (HUMARA), based on the analysis of the methylation status at a polymorphic CAG region in the first exon of the human androgen receptor gene (*AR*). The aim of this study was to evaluate whether the methylation status of the fragile mental retardation protein translational regulator gene (*FMR1*) can provide an XCI pattern similar to that obtained by HUMARA. A set of 48 female carriers of *FMR1* gene normal-sized alleles was examined using two assays: HUMARA and a *FMR1* methylation PCR (mPCR). Ranges were defined to establish the XCI pattern using the methylation pattern of the *FMR1* gene by mPCR. Overall, a 77% concordance of the XCI patterns was obtained between the two assays, which led us to propose a set of key points and a stepwise analysis towards obtaining an accurate result for the XCI pattern and to minimize the underlying pitfalls.

## 1. Introduction

Females are usually less susceptible to recessive pathogenic variants in X-chromosome genes, being asymptomatic with a complete absence of clinical features or showing a phenotype less severe than those males presenting the same pathogenic variants [1]. This is assumed to be due to the X-chromosome inactivation (XCI) phenomena, where preferential inactivation of the X-chromosome with the normal allele results in an incomplete penetrance or in variable clinical presentations [2,3,4,5,6,7,8]. Such an event can complicate risk assessment and genetic counselling in females, such as in the case of *FMR1* full mutation carriers [9]. A skewed XCI pattern may also be observed in the general population, known to increase with the aging process by mechanisms not fully understood, and it has been associated with females with idiopathic premature ovarian insufficiency or recurrent pregnancy loss [10,11,12,13,14,15,16,17,18]. The methylation pattern of the CpG islands adjacent to the polymorphic CAG repeat in the androgen receptor (*AR*) gene located near the X-inactivation center correlates well with the methylation of the X-chromosome [19]. The method described by Allen et al., to analyse the *AR* gene methylation status, designated Human Androgen-Receptor Assay (HUMARA), is considered the gold standard assay to determine the XCI pattern [20]. The frequency of non-informativity, as well as the technical challenges (e.g., allele dropout, presence of stutter bands, among others) inherent to the properties of GC-rich polymorphic regions, justifies the testing of other loci with CpG sites susceptible to inactivation [21]. The CGG repetitive region in the promotor of the fragile mental retardation protein translational regulator (*FMR1*) gene, when expanded to over 200 triplets (full mutation), leads to methylation and gene silencing and, consequently, Fragile X syndrome (FXS; OMIM #3000624) [22,23]. Past studies have assessed the utility of using the *FMR1* locus (Xq27.3) to determine the XCI pattern. Analysis was done by visual inspection of PCR fragments, after digestion with a methylation-sensitive endonuclease [24,25]. Nowadays, there are more appropriate methods to determine the methylation status of expanded *FMR1* alleles (in the context of FXS diagnosis), resorting to capillary electrophoresis coupled with specific software [26,27]. In this study, we used the AmplideX mPCR *FMR1* Kit (mPCR) and aimed to assess the XCI pattern using the *FMR1* gene methylation status in samples with normal-sized alleles, compared to the corresponding pattern obtained using HUMARA. We used samples from females showing random and skewed XCI patterns, which enabled the establishment of intervals for XCI pattern categorization when using the *FMR1* gene. The absence of a complete overlap between the results of both assays led us to propose a set of key points and a stepwise analytical procedure for accurate XCI pattern result assessment, while minimizing the effect of the pitfalls underlying the use of this locus.

## 2. Materials and Methods

### 2.1. Retrospective Study

The results of the X-chromosome inactivation (XCI) pattern category of 74 female samples, with a mean age of 25.2 ± 15.1 years (range 1–68), tested between January 2014 and February 2021 at our laboratory in a diagnostic context, were re-analysed. This data set includes carriers of X-autosome translocation, Fragile-X full mutation, and presumably pathogenic recessive X-linked variants.

### 2.2. Study Cohort

Forty-one females with fertility issues and seven oocyte donors (*n* = 48), with a mean age of 33.8 ± 5.0 years (range 20–40), were selected from a set of females previously recruited at the Centre for Medically Assisted Procreation and Public Gamete Bank, Centro Materno-Infantil do Norte Dr. Albino Aroso (CMIN), Centro Hospitalar Universitário do Porto (CHUPorto), as part of B. Rodrigues’s Ph.D. research project (2020.119/097-DEFI/099-CE). Genomic DNA extracted from peripheral blood samples were used to assess the methylation status of the *FMR1* and *AR* genes, as well as the number of CGG and CAG repeats, respectively.

### 2.3. Molecular Studies

#### 2.3.1. *AR* Gene Methylation Status

The *AR* methylation status was determined using the Human Androgen-Receptor (*AR*) Assay, HUMARA (hereafter Assay A), adapted from Allen et al., [20]. Briefly, 150 ng of DNA was digested with the methylation-sensitive enzyme *Hha*I (New England BioLabs^®^, Ipswich, Massachusetts, EUA) and incubated overnight at 37 °C, followed by 20 min of inactivation at 70 °C. The amplification of the digested and undigested fragments was performed using PCR Master Mix (Promega^®^, Madison, WI, USA) and NED-labelled forward primer, with the following thermal cycling conditions: denaturation of 5 min at 95 °C, 29 cycles of 45 s at 95 °C, 30 s at 62 °C, and 30 s at 72 °C, with a final extension of 15 min at 72 °C. PCR products were resolved on ABI PRISM^®^ 3130*xl* Genetic Analyzer (Applied Biosystems™, Foster City, CA, USA) using 500 ROX™ size standard (Gene Scan™, Warrington, UK) and were analysed using GeneMapper^®^ software version 4.0 (Applied Biosystems™). The percentage of *AR* allele methylation was determined using the area values, normalized to the corresponding undigested allele area. Assay A cut-offs used were previously defined (Supplementary Table S1) [21].

#### 2.3.2. *FMR1* Gene Methylation Status

The *FMR1* promoter methylation status was determined using the AmplideX® mPCR *FMR1* kit (AmplideX^®^ mPCR *FMR1* Kit, Asuragen^®^, Austin, TX, USA) (hereafter Assay B), according to the manufacturer’s instructions [26]. The optimized protocol and components of the kit used a total of 160 ng of DNA and the methylation-sensitive endonuclease *Hpa*II. Undigested fragments were amplified with FAM-labelled primers, whereas digested fragments (after methylation-sensitive *Hpa*II restriction) were amplified using HEX-labelled primers. PCR products were resolved as described above, using a capillary electrophoresis (CE) injection at 2 kV for 5 s, as recommended for normal alleles with a signal intensity beyond the CE instrument saturation limit. This assay determines both the number of CGG repeats and the methylation percentage of each *FMR1* allele. Fragment size and height were determined using GeneMapper^®^ software version 4.0 (Applied Biosystems™). The percentage of the allele methylation status was determined using the ratio of allele height (digested/undigested) and was normalized to the sum of the ratios digested/undigested of each allele.

### 2.4. Statistical Analysis: Reproducibility and Performance Comparison of Assays

Statistical analysis was performed with SigmaPlot version 14.0 (Systat Software® Inc., Chicago, IL, USA) and the Real Statistics Resource Pack for Excel (available at https://www.real-statistics.com/, accessed on 22 December 2021. The Shapiro−Wilk test was used to assess the normality in the retrospective study.

Four samples were arbitrarily selected (http://vassarstats.net/, accessed on 12 September 2021) for each random and skewed category, as established by Assay A. These samples were used to assess the reproducibility of each assay, addressed in the context of the analytical replication of the results (internal reproducibility). The assays were performed in triplicate in three independent experiments.

The Assay B methylation ranges and categories, i.e., random, moderate, and high, were selected to maximize the similarity between the results of both assays.

The internal reproducibility between replicates for each assay was performed with the Brown−Forsythe test, considering the following four XCI patterns: random, moderately skewed, highly skewed, and completely skewed. The Kolmogorov−Smirnov test was used to compare the two assays in terms of the XCI pattern categories. The Chi-square test was used to compare the number of samples (frequency) by XCI pattern categories between the two assays. A significance level of 0.05 was considered for all of the statistical tests.

## 3. Results

### 3.1. Retrospective Analysis of Samples Tested by HUMARA

HUMARA determines the methylation percentage of the *AR* gene alleles and is commonly applied to establish the pattern of XCI. Among the retrospective cases (*n* = 74), a high number of uninformative samples with respect to the XCI pattern and the polymorphic locus (CAG homozygosity) was observed in 10.8% (*n* = 8/74). The informative samples were categorized according to ranges previously defined (Supplementary Table S1) [21]. The majority of the samples (71.2%, *n* = 47/66) showed a random XCI pattern, 15.2% (*n* = 10/66) showed moderately skewed, 1.5% (*n* = 1/66) highly skewed, and 12.1% (*n* = 8/66) completely skewed patterns. In this retrospective analysis, the full spectrum of normally distributed XCI patterns was observed (Shapiro−Wilk test: W = 0.9770, *p* = 0.870; mean [54:46] ± [23:75] and median [55:45]). Borderline results such as [78:22] (*n* = 2/66), [80:20] (*n* = 1/66), [81:19] (*n* = 1/66), [82:18] (*n* = 1/66), [88:12] (*n* = 1/66), and [90:10] (*n* = 3/66), were observed among 13.6% of the informative samples (*n* = 66). When combining the results that were either borderline or uninformative (homozygous), the XCI pattern was not (accurately) defined in around 23% (*n* = 17/74) of the samples. This observation prompted us to analyse if the methylation percentage of another locus, namely the *FMR1* gene, could be used to determine the XCI pattern, thereby reducing the number of inconclusive results.

### 3.2. Establishment of the Intervals for XCI Pattern Determination Using FMR1 mPCR

We determined the methylation status of the *AR* and *FMR1* genes using Assay A and B, respectively, in 48 samples. The XCI patterns were categorized according to the ranges previously defined for Assay A (Supplementary Table S1) [21]. Table 1 shows the ranges used after the Assay B values were adjusted, to minimize the differences between the two assays. Assay A results showed a random XCI pattern in 75% of samples (*n* = 36/48), high skewing in 12.5% (*n* = 6/48), and moderate skewing in 12.5% (*n* = 6/48) (Table 2). Two samples showed borderline results: [19:81] (*n* = 1/48) and [91:9] (*n* = 1/48). Assay B revealed a random XCI pattern in 77% of the samples (*n* = 37/48), 16.7% (*n* = 8/48) had moderate skewing, and only 6.3% (*n* = 3/48) with high skewing (Table 2). Borderline results were obtained in four samples: [73:27] (*n* = 2/48), [90:10] (*n* = 1/48) and [91:9] (*n* = 1/48). For both assays, a random XCI pattern was observed in the majority of the samples, and no case was seen to have complete skewing (>99:1 or >1:99).

### 3.3. Performance Comparison Showed No Differences between the Assays

**Figure 1:**
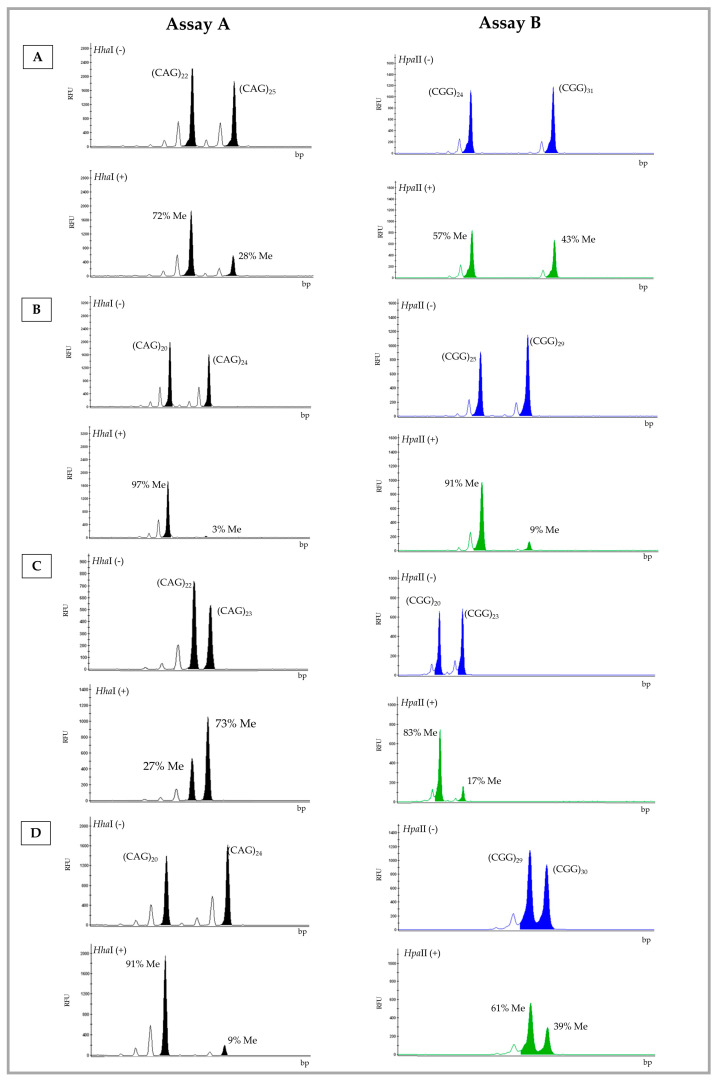
Electropherogram of the A and B Assay results for samples number 29 (**A**), 37 (**B**), 39 (**C**), and 45 (**D**). Digested allele (+) and undigested allele (−). Number of CAG (*AR*) and CGG (*FMR1*) repeats and respective percentage of methylation (Me). Blue (FAM-labelled)—undigested allele; green (HEX-labelled)—digested allele; RFU—relative fluorescence Units; bp—base pairs.

Table 2 summarizes the results obtained after testing 48 samples using the two assays and categorized according to the newly established XCI pattern based on the *FMR1* methylation status. In Figure 1, representative electropherograms of samples number 29 (Figure 1A), 37 (Figure 1B), 39 (Figure 1C), and 45 (Figure 1D) are shown. When the XCI pattern categories were considered, i.e., shape of the distributions per category, no significant differences were found between the assays (Kolmogorov−Smirnov Test: D = 0.333, *p* = 0.976; Table 2). Up to 77% of the samples showed concordant results in both assays: 66% of samples (n = 32/48) with a random pattern, 6.4% (*n* = 3/48) were moderately skewed, and 4.2% (*n* = 2/48) were highly skewed (Supplementary Table S2).

Reproducibility within each assay was evaluated by testing eight samples in triplicate. A significant Brown−Forsythe test result (Assay A: F* = 2089.739, df = 7, *p* < 0.005; Assay B: F* = 1704.674, df = 7, *p* < 0.005) suggested our data did not meet the assumption of homogeneity of variances, and is indicative of a low internal reproducibility.

### 3.4. Small and Large Repeat Number Differences among FMR1 Alleles Explain Distinct XCI Pattern Categorizations

We investigated whether samples showing small differences (one or two repeats) in *FMR1* allele sizes could fall into distinct categories when comparing the results of both assays. In fact, in four samples where *FMR1* alleles differed by less than two repeats, the XCI pattern category was distinct among the assays (samples number 40, 42, 45, and 47; Figure 1D and Supplementary Table S2). We further speculated that a large difference in the number of repeats among *FMR1* alleles (≥7 CGGs) might also contribute to distinct XCI pattern categorizations, due to the preferential amplification of the smaller allele. This was observed in five cases (Supplementary Table S2; samples number 38, 41, 44, 46, and 48). Borderline results, such as that of sample 39 (Figure 1C and Supplementary Table S2), also led to the distinct categorization of the XCI pattern among the two assays. We speculate that the cumulative effect of preferential amplification of the smaller allele and inaccuracy introduced due to the presence of stutter bands, particularly in samples with small differences in allele repeat numbers (≤2), may influence the quantification of the methylation percentage and consequently the XCI pattern.

## 4. Discussion

In the current study, we compared the results of 48 females using *FMR1* methylation status and HUMARA in order to establish the *FMR1* XCI pattern range limits. Our results revealed 77% agreement regarding the XCI pattern categorization between the two assays. Furthermore, analysis of the discordant samples led us to suggest a set of key points that should be taken into consideration when evaluating the XCI pattern using the percentage of *FMR1* methylation. In samples where alleles differed by up to two trinucleotide repeats, determination of the XCI pattern might be challenging. We also observed that when the stutter band from the larger allele, with a similar amplification intensity, overlapped with the primary band of the smaller allele, it interfered with the calculation of the area of the true allele, hampering an accurate XCI categorization. Previous reports suggest that inaccurate XCI pattern categorization might occur in samples with *AR* alleles differing by ≤2 repeats, due to difficulty in discriminating the true allele area value from the overlapping stutter bands [21,28]. In samples where this is observed, the XCI pattern should not be based on the obtained methylation pattern. In alleles that differ by ≥7 repeats, the use of *FMR1* allele methylation percentage might also become problematic due to amplification bias. In order to minimize the putative preferential amplification of the smaller allele, we suggest testing these samples in triplicate, as our analysis showed that the use of mean results of replicates was more accurate even in cases of low internal reproducibility. We suggest that these key-points should also be considered when using methylation of other X-linked loci by any methodology. Nevertheless, further studies, such as an increased number of test samples, are warranted. In fact, one of the major limitations of our study is the small number of samples, as well as the trial-and-error process used to establish the *FMR1*-based XCI pattern categories, to maximize the number of samples that matched the HUMARA XCI categories.

Furthermore, we cannot exclude that the mechanism of epigenetic control of the *FMR1* gene is different from that of the *AR* gene. This could be the reason underlying the distinct results observed in cases with high skewing obtained by Assay A, which is not reproduced in Assay B. Of note, the methylation status of the *FMR1* normal alleles was determined using a commercial mPCR kit designed to determine the percentage of methylation of the expanded alleles with the purpose of diagnosing Fragile-X syndrome. Other methodologies or methylation-sensitive enzymes should also be tested. In addition, other candidate genes for further additional inactivation tests could be of interest, particularly those previously described [29,30]. Nevertheless, in might be difficult to find a gene that completely mirrors the methylation status of the *AR* gene, allowing for the extrapolation of the X-chromosome methylation pattern. Although the large majority of the X-chromosome genes are subjected to methylation, about 15% escaped XCI [31,32]. Furthermore, an incomplete correlation between XCI pattern and the expression of the *AR* gene was reported. The discrepancy between *AR* locus methylation and *AR* expression was observed in healthy females with a skewed XCI pattern, suggesting that the CpG methylation did not extend to the promoter or at least was unable to inhibit the *AR* promotor [33]. This could also explain why in some samples, XCI skewing was observed using Assay A and not Assay B (Supplementary Table S2; Sample number 47). Future expression studies should be undertaken to support this hypothesis.

We suggest the use of the *FMR1* methylation status to infer the XCI pattern, after triplicate testing, when (i) alleles differ by more than two and less than seven CGG repeats, (ii) in the absence of amplification bias (maximum difference between undigested allele area/height of: 1:1.3 or 1.3:1), and (iii) the result is established outside borderline (±2) limits. More studies are needed to further increase the power of the statistical analysis and to validate the ranges established for each pattern category, as well as the expression analysis of both *AR* and *FMR1* genes. Nevertheless, this study shows that the *FMR1* gene methylation mirrors the methylation of the *AR* gene and can be used to determine the XCI pattern, with a particular impact on samples where *AR* locus homozygosity is observed. Our results warrant a new line of investigation to further increase the knowledge of the epigenetic mechanisms that regulate XCI and the methylation of *FMR1* and *AR* genes.

## 5. Conclusions

This study shows that *FMR1* gene methylation mirrors the methylation of the *AR* gene and can be used to determine the XCI pattern, with a particular impact in samples where *AR* locus homozygosity is observed. Our results warrant a new line of investigation to further increase the knowledge of the epigenetic mechanisms that regulate XCI and the methylation of *FMR1* and *AR* genes.

## Figures and Tables

**Table 1:** Categories established for XCI pattern determination using *FMR1* mPCR (Assay B).

	Assay B			
XCI Pattern Categories	Random	Moderately Skewed	Highly Skewed	Completely Skewed
Ranges	[25:75–75:25]	[11:89–25:75[	[1:99–11:89[	[0:100–1:99[
	[75:25–25:75]	]75:25–89:11]	]89:11–99:1]	]99:1–100:0]

**Table 2:** Summary of the results obtained in the forty-eight samples.

	XCI Pattern Categories		
Assay	Random	Moderately Skewed	Highly Skewed
	*n* (%)	*n* (%)	*n* (%)
A	36 (75%)	6 (12.5%)	6 (12.5%)
B	37 (77%)	8 (16.7%)	3 (6.3%)
Partial χ^2^ (df = 1)	0.014	0.286	1.00
*p* value *	0.907	0.593	0.317

* Chi-square value calculation was based on absolute frequencies in each category (χ^2^ = 1.299; df = 2; *p* = 0.522); *n* = Number of samples in the category; % = Percentage of samples in the category.

## Data Availability

Data are contained within the article or supplementary material.

## Supplementary Materials

The following are available online at https://www.mdpi.com/article/10.3390/genes13030419/s1. Table S1: References ranges used for X-chromosome inactivation pattern determination by HUMARA (Assay A). Table S2: Summary of the results of *AR* (Assay A) and *FMR1* (Assay B) genes methylation pattern.
