# Supplementary material for: Use of the FMR1 Gene Methylation Status to Assess the X-Chromosome Inactivation Pattern: A Stepwise Analysis
Source: Genes (Basel). 2022 Feb 25;13(3):419. doi: 10.3390/genes13030419 (PMC8951761; doi:10.3390/genes13030419)
Supplement: Supplementary file 1 [file genes-13-00419-s001.zip › genes-1600741-supplementary.pdf]

Supplementary Tables

Table S1. References ranges used for X-chromosome inactivation pattern determination by HUMARA (Assay A).

	Assay A			
**XCI pattern categories**	Random	Moderately skewed	Highly skewed	Completely skewed
**Ranges***	[20:80-80:20]<br>[80:20-20:80]	[10:90-20:80[<br>]80:20-90:10]	[1:99-10:90[<br>]90:10-99:1]	[0:100-1:99[<br>]99:1-100:0]

*Ranges were previously defined by Amos-Landgraf et al., [21].

Table S2. Summary of the results of *AR* (Assay A) and *FMR1* (Assay B) genes methylation pattern. In the material and method section, the calculations on the X-chromosome inactivation (XCI) pattern and respective category on the basis of the percentage (%) of methylation of each gene are explained.

	Assay A			Assay B			
Sample number	% methylation Allele 1	% methylation Allele 2	XCI pattern	% methylation Allele 1	% methylation Allele 2	XCI pattern	$(CGG_n)_{allele\ 2}$ - $(CGG_n)_{allele\ 1}$
**1**	48	52	Random	44	56	Random	7
**2**	42	58	Random	30	70	Random	28
**3**	56	44	Random	34	66	Random	2
**4**	71	29	Random	36	64	Random	1
**5**	70	30	Random	57	43	Random	3
**6**	65	35	Random	32	68	Random	11
**7**	50	50	Random	49	51	Random	12
**8**	27	73	Random	37	63	Random	1
**9**	63	37	Random	41	59	Random	11
**10**	60	40	Random	48	52	Random	2
**11**	56	44	Random	49	51	Random	13
**12**	37	63	Random	69	31	Random	5
**13**	48	52	Random	56	44	Random	7
**14**	55	45	Random	40	60	Random	13
**15**	28	72	Random	35	65	Random	10
**16**	41	59	Random	58	42	Random	10
**17**	63	37	Random	45	55	Random	6
**18**	55	45	Random	38	62	Random	6
**19**	66	34	Random	69	31	Random	3
**20**	40	60	Random	48	52	Random	1
**21**	58	42	Random	43	57	Random	14
**22**	72	28	Random	40	60	Random	11
**23**	32	68	Random	44	56	Random	1
**24**	47	53	Random	59	41	Random	2
**25**	43	57	Random	56	44	Random	9
**26**	59	41	Random	59	41	Random	10
**27**	44	56	Random	45	55	Random	9
**28**	64	36	Random	44	56	Random	11
**29**	72	28	Random	57	43	Random	7
**30**	35	65	Random	73	27	Random	23
**31**	29	71	Random	28	72	Random	2
**32**	23	77	Random	73	27	Random	5
**38**	71	29	Random	17	83	Moderately skewed	11
**39**	27	73	Random	83	17	Moderately skewed	3
**40**	41	59	Random	82	18	Moderately skewed	1
**41**	52	48	Random	79	21	Moderately skewed	7
**33**	82	18	Moderately skewed	79	21	Moderately skewed	13
**34**	83	17	Moderately skewed	22	78	Moderately skewed	16
**35**	83	17	Moderately skewed	15	85	Moderately skewed	10
**42**	83	17	Moderately skewed	32	68	Random	1
**43**	19	81	Moderately skewed	35	65	Random	3
**44**	83	17	Moderately skewed	97	3	Highly skewed	7
**45**	91	9	Highly skewed	61	39	Random	1
**46**	93	7	Highly skewed	81	19	Moderately skewed	9
**47**	93	7	Highly skewed	28	72	Random	1
**48**	94	6	Highly skewed	72	28	Random	7
**36**	93	7	Highly skewed	91	9	Highly skewed	1
**37**	97	3	Highly skewed	90	10	Highly skewed	4

n = number of CGGs
